# Robust Stereo Visual Odometry Using Improved RANSAC-Based Methods for Mobile Robot Localization

**DOI:** 10.3390/s17102339

**Published:** 2017-10-13

**Authors:** Yanqing Liu, Yuzhang Gu, Jiamao Li, Xiaolin Zhang

**Affiliations:** 1 Bio-Vision System Laboratory, Shanghai Institute of Microsystem and Information Technology, Chinese Academy of Sciences, Shanghai 200050, China; lyq@mail.sim.ac.cn (Y.L.); gyz@mail.sim.ac.cn (Y.G.); jmli@mail.sim.ac.cn (J.L.); 2 University of Chinese Academy of Sciences, Beijing 100049, China

**Keywords:** visual odometry, stereovision, robust estimation, motion estimation, RANSAC

## Abstract

In this paper, we present a novel approach for stereo visual odometry with robust motion estimation that is faster and more accurate than standard RANSAC (Random Sample Consensus). Our method makes improvements in RANSAC in three aspects: first, the hypotheses are preferentially generated by sampling the input feature points on the order of ages and similarities of the features; second, the evaluation of hypotheses is performed based on the SPRT (Sequential Probability Ratio Test) that makes bad hypotheses discarded very fast without verifying all the data points; third, we aggregate the three best hypotheses to get the final estimation instead of only selecting the best hypothesis. The first two aspects improve the speed of RANSAC by generating good hypotheses and discarding bad hypotheses in advance, respectively. The last aspect improves the accuracy of motion estimation. Our method was evaluated in the KITTI (Karlsruhe Institute of Technology and Toyota Technological Institute) and the New Tsukuba dataset. Experimental results show that the proposed method achieves better results for both speed and accuracy than RANSAC.

## 1. Introduction

Mobile robot localization is a fundamental challenge for autonomous mobile robots navigation. A robot needs to know its position to accomplish autonomous navigation. Different sensors and techniques have been used to achieve robot localization, such as global navigation satellite system (GNSS), inertial navigation system (INS), and vision-based localization. Each method has its advantages and disadvantages. GNSS is a very common method for localization by reason of its absolute position without error accumulation, but its accuracy is highly affected by buildings, trees and weather situations, and it’s even not available for indoor situations. INS is fast but has highly accumulated drift, and a highly precise INS is expensive for mobile robots as commercial purposes. Vision-based localization methods have received an increasing interest in the robot vision community because vision-based localization methods provide accurate estimation of camera motion along with information for other vision tasks, such as obstacle and road detection. Visual odometry (VO) and visual simultaneous localization and mapping (V-SLAM) are two methods of vision-based localization. V-SLAM obtains a global estimation of camera ego-motion through map tracking and loop-closure detection, while VO aims to estimate camera ego-motion incrementally and optimize potentially over a few frames. VO cares about local consistency of the trajectory, whereas V-SLAM is concerned with the global trajectory consistency. Monocular, stereo, omnidirectional and RGB-D (RGB-depth) cameras are the main sensors for VO and V-SLAM. Monocular cameras are cheap and easily deployed, but suffering from scale uncertainty. Omnidirectional cameras can provide more information with a wide field of vision (FOV), while they have a complex system with camera calibrating problems. RGB-D cameras use structured light to estimate depth even in areas with poor visual texture, but they are range-limited by their projectors. Stereo cameras, which have been widely used in VO systems, capture a pair of RGB images at the same moment, thus the image scale can be retrieved by a given baseline.

The remainder of this paper is structured as follows. Section 2 introduces the related work on visual odometry. Proposed methods for robust visual odometry are described in Section 3. Section 4 summarizes the experimental evaluation. Finally, Section 5 concludes the paper.

## 2. Related Work

Visual odometry uses continuously iteration to estimate the ego-motion of the camera frame by frame. Methods of existing visual odometry can be generally classified into two classes: feature-based methods and direct methods. Feature-based methods [1,2,3,4] detect a set of salient image features (e.g., points, lines) in each image and track them in consecutive frames with feature descriptors. The ego-motion is estimated by using the epipolar geometry, which can be finally refined by minimizing the reprojection errors. In contrast, direct methods [5,6,7] estimate the ego-motion directly from the intensity differences between consecutive images. Using visual sensors input alone for the estimation of a vehicle’s ego-motion started in the early 1980s and was introduced by Moravec [8]. Most of the early research in visual odometry was driven by the NASA (National Aeronautics and Space Administration) Mars exploration program, which aimed to measure the ego-motion of planetary rovers in Mars with wheel slippage in uneven and rough terrains. Since the work of Nister et al. [1], visual odometry has received an increasing interest. They proposed a feature-based visual odometry system that provided real-time ego-motion estimation for navigational purposes with monocular and stereo cameras. After that, feature-based VO algorithms basically have these steps:

- Detect features in each image.
- Match them in two consecutive frames and remove the wrong matches.
- Estimate the ego-motion of the cameras.

Many improvements have been made in those aspects of visual odometry. Feature detectors for real-time algorithms including Harris corners [9], FAST (Features from Accelerated Segment Test) [10] Shi–Tomasi [11] and blob detectors have been widely used for VO. Evaluation of feature detectors and descriptors has been described for indoor VO in [12] and outdoor environments in [13]. Besides the point features, line features are also added to estimate the ego-motion in [14]. Because there are many scenes where the points matching is unreliable while the lines are well matched by multi-pixel support [15]. In [16], the authors proposed a novel method of adopting the whole history of tracked feature points. Features tracked over past frames are integrated into one single feature point and the estimation of ego-motion is improved by reducing the drift error.

Feature matching is an important step for visual odometry, which searches for corresponding features in other images. Feature descriptors are compared by using a similarity measure. Instead of comparing all feature descriptors, many VO algorithms perform a constrained matching, which only searches for features near a given feature. This can be done by applying a motion model or epipolar matching. Feature tracking is also a method to find correspondences between consecutive frames. Optical flow methods such as KLT (Kanade–Lucas–Tomasi) [17] and dense scene flow [18] have also been employed.

The motion estimation can be generally divided into three methods: 2D to 2D, 3D to 3D and 3D to 2D, depending on the feature correspondences. As described in [1], motion estimation using 3D to 2D is more accurate than 3D to 3D. 3D to 2D computes the motion by minimizing the image reprojection error, which can avoid the uncertainty in the depth direction.

The data fusion of visual odometry with other sensors like GPS (Global Positioning System), lasers, barometric sensors and IMU (Inertial measurement unit) can improve the accuracy of the motion estimation, which can be used for state estimation of Micro-Aerial Vehicle (MAV) as described in [19].

To improve the robustness of visual odometry, RANSAC has been a standard method for model estimation in the presence of outliers. In [20], RANSAC is used to reject outliers caused by moving objects in dynamic environments. To the best of our knowledge, a few improvements have been made in RANSAC for visual odometry. The most popular ones are Preemptive RANSAC [21] and PARSAC [22]. Preemptive RANSAC [21] is popular for its advantages in real-time applications. This method is mainly based on preemptive scoring of hypotheses and a fixed number of iterations. Preemptive scoring avoids excessive scoring of bad hypotheses contaminated by outliers or noise, which makes the scoring procedure more efficient on a limited time budget. Most recently, a novel prior-based adaptive RANSAC (PARSAC) is described in [22], which is efficient to remove outliers when the inlier ratio is rather low in AR and indoor applications. The authors observed that static background feature points usually distributed evenly, whereas the dynamic feature points aggregated in a few small textured areas. Feature points were sampled as even as possible in the whole image. They used inlier/outlier distribution information from previous frames as prior information to guide the sampling of the current frame. PARSAC has good performance when facing large amounts of dynamic feature points, but it is mainly used in AR applications. Preemptive RANSAC only cares a lot about improving the speed of RANSAC but not the accuracy. Our method is partly inspired by these two methods. The hypotheses are generated by prior information just like PARSAC, but we use features’ ages and similarities instead of inlier/outlier distribution. The evaluation of hypotheses is also very fast by SPRT. With these improvements, our method is very suitable for mobile robot localization in indoor and outdoor environments.

In this work, we propose a robust visual odometry using an improved RANSAC-based method, named PASAC (Priori-based Aggregation SAmpling Consensus). A standard feature-based visual odometry framework is adopted in our method. We concentrate on the generation and the evaluation of hypotheses, which makes the process much faster than standard RANSAC. To improve the accuracy, we propose an aggregation strategy to aggregate the best hypotheses. Thus, our method is faster and more accurate than standard RANSAC, which is very suitable for real time applications like visual odometry.

## 3. Proposed Method

**Figure 1:**
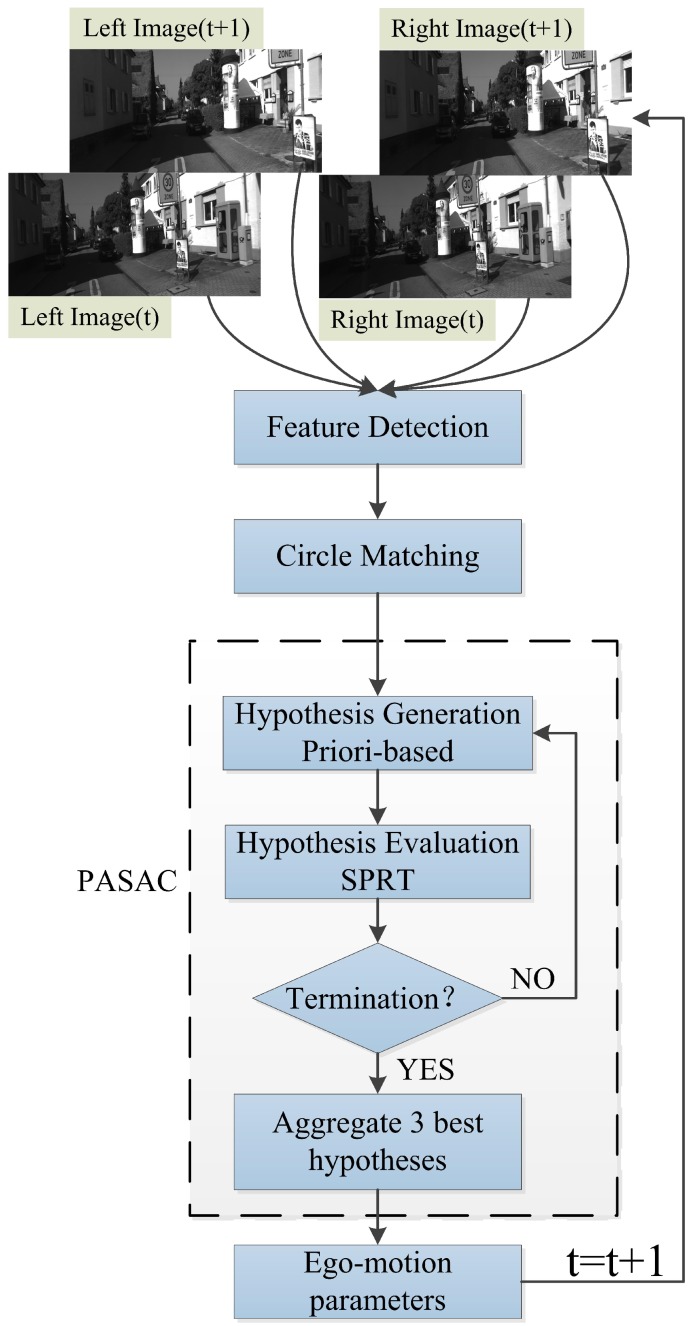
Algorithm architecture, camera images are from the KITTI dataset. The dash rectangle is our robust motion estimation method PASAC.

In this section, we describe our proposed method in detail. We follow a standard feature-based VO pipeline. Our proposed method is illustrated in Figure 1. Firstly, we use a pair of calibrated and rectified stereo images as our input data. Feature points are detected and descriptors are extracted from both left and right images. After stereo matching, the 3D position of feature points can be calculated by using calibration parameters of the stereo cameras. When the next pair of images comes, we perform a circle matching between left and right images and two consecutive images. Outliers caused by mismatching between stereo and consecutive images can be rejected through circle matching, after which we use our robust motion estimation method PASAC. To reduce the impact of outliers, a minimum sample is not totally randomly generated. We generate the hypotheses on the order of the features’ ages and similarities. Features with larger ages and higher similarities are selected first, which makes our process of hypothesis generation faster and more effective than standard RANSAC. Then, we employ SPRT to perform a faster evaluation process to discard bad hypotheses in advance. After hypothesis evaluation, we aggregate the three best hypotheses to improve the accuracy of RANSAC. These steps make our robust motion estimation much faster and more accurate than standard RANSAC. In the next sections, we describe each step of our robust visual odometry in detail.

### 3.1. Robust Feature Detection

**Figure 2:**
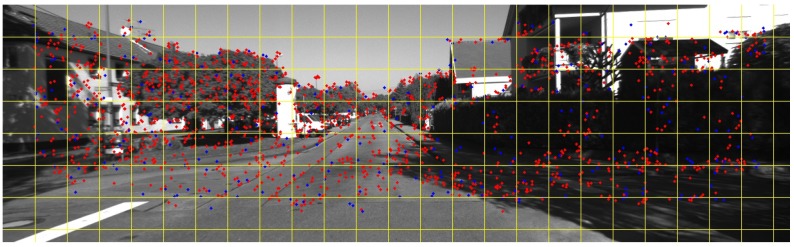
Features before and after bucketing: the red points stand for features before bucketing, the blue points stand for features after bucketing, and the yellow lines depict individual buckets.

Feature-based VO starts from feature detection. We detect the corner-like feature points in the left and right images of the current frame utilizing blob and corner masks as described in [23]. After the key points are detected, we employ a non-maximum suppression algorithm [24]. The descriptors are extracted by the 3×3 Sobel operator. Then, the descriptors in the left and right images are matched by the sum of absolute differences (SAD), during which we also use the epipolar constraint to remove some outliers. Lastly, a subset of features is chosen by feature bucketing [20]. Figure 2 shows the feature points detected before and after bucketing. In this way, we use less features to compute the ego-motion and reduce the computational complexity. At the same time, the features are uniformly distributed over the whole image, which reduces the drift rates of the feature-based VO. Using the correspondence between the remaining key points in the left and right images, we can calculate the disparity of key points and the 3D position of each key point can be obtained.

### 3.2. Feature Circle Matching

**Figure 3:**
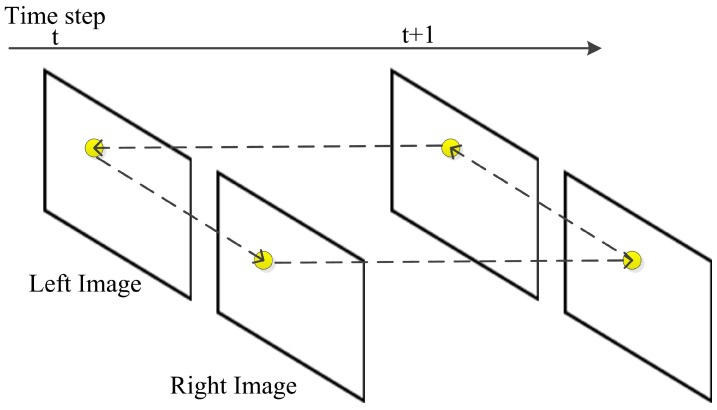
Circle matching. Matching the features from time t in the left image, the right image next, then time t + 1 in the right image, and finally the left image. A feature is selected when it is matched in all four images.

The circle matching is used to reject outliers caused by mismatching between stereo and consecutive images. When getting two pairs of images from time step t and t + 1, we start matching from the previous left image, the previous right image next, then the current right image, and finally the current left image. Once a feature is matched in all frames, the circle matching is achieved, as shown in Figure 3. Otherwise, the feature is rejected if the circle is not closed. When the circle is closed, an additional check is performed with normalized cross correlation (NCC) on a 15×15 pixels patch around the feature position. NCC is much slower but more reliable than SAD, so we only use NCC as a double check after SAD.

### 3.3. Feature Tracking

**Figure 4:**
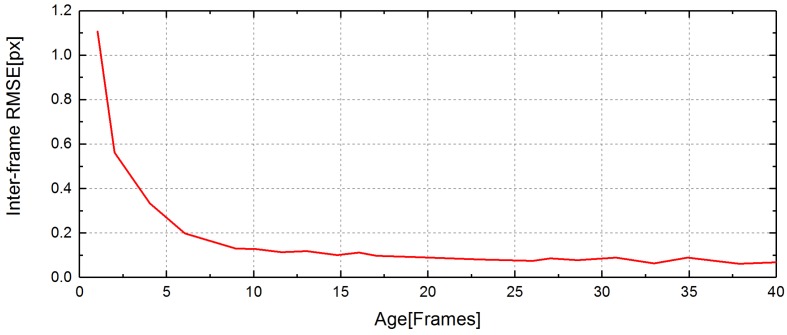
RMSE (root-mean-square error) of inter-frame feature position as a function of the survival age. The RMSE is lower if the feature’s age is bigger.

We assign each feature a unique ID in each frame. Then, we record the age of the feature. A feature’s age is increased by one if it is tracked in the current frame. The reason that we record the age of the feature is mainly based on [16]. The feature tracking process is repeated between consecutive images and every tracking step adds a cumulative error to the feature position. In [16], the authors make a statistical analysis of the feature tracking error. They compute the distribution of the accumulated projection error function of the survival age of the feature point as shown in Figure 4. What is shown more specifically in Figure 4 is that features tracked longer have smaller errors and are more likely to be inliers. The reason is that possessing some properties makes them easier and more accurate to be tracked. Those features with larger ages should be chosen first, which is used in later sections.

### 3.4. Robust Motion Estimation

#### 3.4.1. Modeling

Let $x_{s,i}^{d}={\left[u_{s,i}^{d},v_{s,i}^{d}\right]}^{T}\in R^{2},d\in \left\{l,r\right\},s\in \left\{1,2,\ldots ,t,t+1,\ldots \right\},i\in \left\{1,2,\ldots ,n\right\}$ denote the $i^{th}$ feature point in time step *s* in left image *l* or right image *r*. Let $X_{s,i}^{d}={\left[x_{s,i}^{d},y_{s,i}^{d},z_{s,i}^{d}\right]}^{T}$ denote the 3D position of the feature points. It can be calculated through the pinhole camera model and stereo matching, i.e., Equation (1), where f is the focal length, ${\left[c_{u},c_{v}\right]}^{T}$ is the image principal point, and [R|t] are rotation and translation between pixel coordinate and physical coordinate:

(1) $$\lambda \left(\begin{array}{c} u\\ v\\ 1 \end{array}\right)=\left(\begin{array}{ccc} f & 0 & c_{u}\\ 0 & f & c_{v}\\ 0 & 0 & 1 \end{array}\right)\left[\begin{array}{c} R|t \end{array}\right]\left(\begin{array}{c} x\\ y\\ z\\ 1 \end{array}\right).$$

The ego-motion change of the cameras from time t−1 to time *t* is given by the rotation matrix R∈SO(3) and 3D translation vector $T={\left[t_{x},t_{y},t_{z}\right]}^{T}\in R^{3}$. The 3D feature points calculated from the previous view are projected onto the image plane of the current view through:

(2) $$\pi^{l}\left(X_{t-1}^{l};R,t\right)=\left(\begin{array}{ccc} f & 0 & c_{u}\\ 0 & f & c_{v}\\ 0 & 0 & 1 \end{array}\right)\left[\begin{array}{c} R|t \end{array}\right]\left(\begin{array}{c} x_{t-1}^{l}\\ y_{t-1}^{l}\\ z_{t-1}^{l}\\ 1 \end{array}\right),$$

where π is the reprojection function, and [R|t] are rotation and translation between two views. Then, the cost function can be formulated by the image reprojection error through Equation (3), where *N* is the total number of the feature points:

(3) $$\sum_{i=1}^{N}\left|\right|x_{t,i}^{l}-\pi^{l}\left(X_{t-1}^{l};R,t\right){\left|\right|}^{2}+\left|\right|x_{t,i}^{r}-\pi^{r}\left(X_{t-1}^{r};R,t\right){\left|\right|}^{2}.$$

The parameters of ego-motion can be calculated by minimizing the cost function. We use the LM (Levenberg–Marquard) algorithm [25] to solve this non-linear least squares optimization problem iteratively.

#### 3.4.2. Hypothesis Generation

Since feature detection and feature matching are not perfect, the matches can be incorrect or inaccurate. After circle matching, outliers will always exist and degrade VO accuracy, and sometimes even make the algorithm fail. Outliers are generally dealt with by using Random Sample Consensus (RANSAC) [26]. The RANSAC algorithm is simple but powerful, which is very commonly employed for estimating the parameters of a model with outliers. RANSAC operates in a hypothesize-and-verify framework. It starts with a randomly minimal sampling and estimates the model parameters from the subset of sampled points. Then, the model is evaluated on the entire data set, which separates the data from inliers and outliers with a given threshold. The operation is repeated unless the probability of finding a model better than the current best model falls below a given threshold. Standard RANSAC needs to explore *k* hypothesis in order to find at least one outlier-free consensus set with confidence $\eta_{0}$, through:

(4) $$k\geq \frac{log(1-\eta_{0})}{log(1-\epsilon^{m})},$$

where ϵ is the percentage of inliers in the data set, and *m* is the minimum sample size. This indicates that the capability of RANSAC dealing with the data contaminated with large outlier ratio degrades. Thus, it is better to generate good hypotheses first.

**Figure 5:**
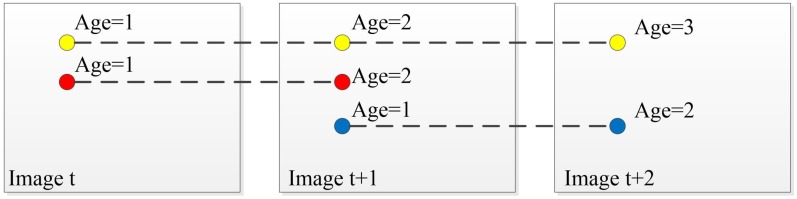
The feature’s age is increased by one if it is tracked in the current frame. The yellow point should be chosen first in Image t+2.

The hypotheses are generated by uniformly sampling the input data set in a standard RANSAC algorithm. This is mainly because no priori information about the input data set is available. However, in the specific applications, that priori information is always available makes it possible to be used to generate better hypotheses. According to this, we propose a method to generate hypotheses based on Progressive Sample Consensus (PROSAC) [27]. The correspondences between stereo and consecutive images are obtained by a similarity function of feature descriptors. As described in [27], points with high similarity are more likely to be inliers, and it is better to generate hypotheses by sampling from a reduced set of points with the highest similarity. In Section 3.3, we point out that the age of the feature points is related to the cumulative tracking error. Features that die earlier have a larger projection error. Thus, we also consider the age of features when we generate the hypothesis. The correspondence points $u_{i},\;u_{j}$ are sorted in the descending order of features’ ages $A\left(u_{i}\right),A\left(u_{j}\right)$ and similarities scores $q\left(u_{i}\right),q\left(u_{j}\right)$ if the ages are the same, which is described in Equation (5). Figure 5 shows that a feature’s age is increased by one if it is tracked in the current frame. For example, the yellow point’s age is 3 when time is t + 2 because it is tracked in three frames. When selecting features in frame t + 2, we should choose the yellow point first. Then, we use a non-uniform sampling from the sorted sequence. Correspondences with old ages and high similarities are chosen earlier. Finally, Equation (3) is minimized by the LM algorithm to generate hypotheses:

(5) $$u_{i},u_{j}\in U_{N}:i<j\Rightarrow \left\{\begin{array}{cc} A(u_{i}) & >A(u_{j}),\\ q(u_{i}) & >q\left(u_{j}\right)\;if\;A\left(u_{i}\right)=A\left(u_{j}\right). \end{array}\right.$$

#### 3.4.3. Hypothesis Evaluation

After the hypotheses are generated, evaluations are performed over the entire data set in standard RANSAC, and the best hypothesis with the largest consensus set is returned. The computation time of RANSAC can be expressed as:

(6) $$t=k(t_{H}+m_{E}t_{E}),$$

where *k* is the number of hypotheses, $m_{E}$ is the number of points needed to be verified in a hypothesis, and $t_{H}$ and $t_{E}$ denote the time required to compute a hypothesis, and to verify a single point in a hypothesis, respectively. In standard RANSAC, $m_{E}$ equals *N*, where *N* is the number of data points. That is to say, a hypothesis is verified against all data points. However, most hypotheses are “bad” and it is often possible to discard bad hypotheses early in the evaluation process. The evaluation process is sped up by verifying only a few points to discard a bad hypothesis.

We adopt the sequential probability ratio test (SPRT) to discard “bad” hypotheses as described in [28]. In SPRT, the evaluation step is dealt with as an optimization problem, which aims to figure out whether a hypothesis is good $\left(H_{g}\right)$ or bad $\left(H_{b}\right)$ and simultaneously minimize the number of verified points of each hypothesis. SPRT is based on the likelihood ratio:

(7) $$\lambda_{j}=\prod_{r=1}^{j}\frac{p(x_{r}|H_{b})}{p(x_{r}|H_{g})}=\lambda_{j-1}\cdot \frac{p(x_{j}|H_{b})}{p(x_{j}|H_{g})},$$

where $x_{r}$ is equal to 1 if the *r*th data point is consistent with the hypothesis, and $x_{r}$ equals 0 otherwise. $p(1|H_{g})$ denotes the probability that a data point is consistent with a good model can be approximated by the inlier ratio. Instead, $p(1|H_{b})$ can be modeled by a Bernoulli distribution if with a bad model. We separate the data points from outliers and inliers by the reprojection error of the feature points. The reprojection error is calculated by the symmetric transfer error, which can be expressed as:

(8) $$e\left(i\right)={∥x_{t,i}^{l}-T_{H}\left(X_{t-1,i}^{l}\right)∥}^{2}+{∥x_{t,i}^{r}-T_{H}\left(X_{t-1,i}^{r}\right)∥}^{2}+{∥x_{t-1,i}^{l}-T_{H}^{-1}\left(X_{t,i}^{l}\right)∥}^{2}+{∥x_{t-1,i}^{r}-T_{H}^{-1}\left(X_{t,i}^{r}\right)∥}^{2},$$

where $T_{H}$ is the ego-motion transformation between time *t* and t−1, while $T_{H}^{-1}$ is between time t−1 and *t*. Then, we use a given threshold $\delta_{d}$ to separate the data points:

(9) $$\left\{\begin{array}{c} \;e\left(i\right)\geq \delta_{d}\;\;i^{th}\;point\;is\;outlier,\\ e\left(i\right)<\delta_{d}\;\;i^{th}\;point\;is\;inlier. \end{array}\right.$$

The process of SPRT is shown in Algorithm 1. The method begins by verifying whether a point is consistent with the hypothesis through Equation (9). Then, the likelihood ratio λ is computed and compared with *A*: If λ > *A* the hypothesis is “bad” (line 3), if not, it is “good” when *j* is increased to equal *N* (the number of correspondences). *A* is the threshold of SRPT. The procedure of hypothesis generation is terminated when the probability η of missing a set of inliers, larger than the largest support found so far, falls under a predefined threshold $\eta_{0}$. More details are referred to [28].

**Algorithm 1:** The adaptive SPRT
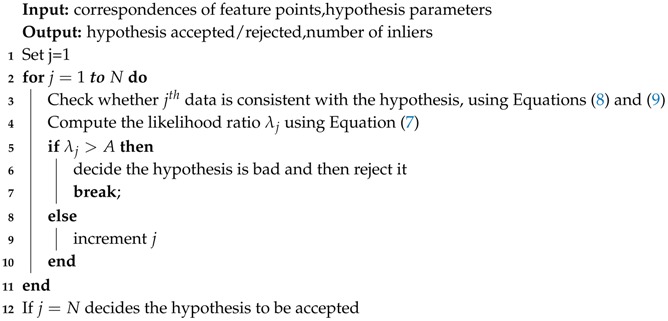


#### 3.4.4. Hypothesis Aggregation

In the process of hypotheses generation and evaluation, we focus on the improvement of the speed of RANSAC. In order to improve the accuracy of RANSAC, we proposed an aggregation strategy inspired by [29]. In standard RANSAC, the best hypothesis with the largest consensus is selected as the final solution. Most hypotheses are discarded though they have large consensus. However, these hypotheses can be very useful to obtain a more accurate hypothesis, on which the idea of hypothesis aggregation is mainly based.

**Figure 6:**
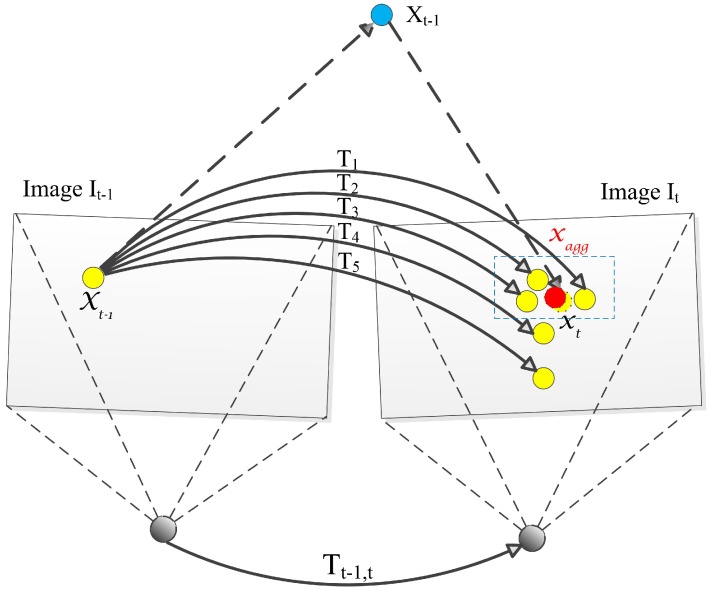
Hypothesis aggregation. Three best hypotheses in the blue dashed rectangle are aggregated, thus the aggregated point $x_{agg}$ is closer to the real matching point $x_{t}$.

Let us consider a feature point $x_{t-1}$ from image $I_{t-1}$ and its real corresponding point $x_{t}$ from image $I_{t}$. At the $k^{th}$ iteration of RANSAC, the hypothesis $H_{k}$ is generated by sampling the matched feature points as described above. Then, $\hat{x_{t}}=\pi \left(X_{t-1}\right)$ is the reprojection of the 3D point $X_{t-1}$ into the image $I_{t}$ according to the hypothesis $H_{k}$. If the hypothesis $H_{k}$ is good enough, $\hat{x_{t}}$ will be close to the real matching point $x_{t}$. After many iterations, hypotheses which make $\hat{x_{t}}$ closer to $x_{t}$ will be found. As illustrated in Figure 6, the ground truth corresponding point $x_{t}$ (dashed yellow point) is the reprojection of the 3D point $X_{t-1}$ (the blue point) into the image $I_{t}$ according to the ground truth transformation $T_{t-1,t}$, while other yellow points are calculated according to the estimated transformations $T_{1}$ to $T_{5}$. $T_{1}$ to $T_{5}$ means five transformations calculated by hypotheses $H_{1}$ to $H_{5}$. It is obvious that $T_{1}$ is the closest to the ground truth transformation because the reprojection of $X_{t-1}$ is the closest to the real corresponding point $x_{t}$ , while $T_{5}$ is the farthest from the real transformation. The accuracy of these hypotheses is affected by the underlying noise of the model hypothesis and the measurement noise of the input data. Both of the two noises have zero mean and a symmetric distribution function, which makes the process of hypothesis aggregation yield a value close to the true corresponding point $x_{t}$. In Figure 6, the three best feature points in blue dash rectangles are aggregated, and the aggregated point $x_{agg}$ is closer to the real matching point $x_{t}$.

More generally, let ${\left\{x_{t-1,i},x_{t,i}\right\}}_{i=1,\ldots ,N}$ be a set of corresponding points from consecutive images $I_{t-1}$ and $I_{t}$. For each iteration *k* with hypothesis $H_{k}$ , the estimated reprojection points are denoted as:

(10) $${\hat{x}}_{t,i}=H_{k}\left(X_{t-1}\right).$$

In order to reflect the reliability of hypotheses during the process of hypothesis aggregation, we assign a weight for each generated hypothesis, which will make the aggregated point have a high probability closer to its real value $x_{t}$. The weights have already been calculated by the process of hypotheses evaluation by Equation (8). Thus, the weight of hypothesis *k* can be expressed as:

(11) $$\omega_{k}=\sum_{i=1}^{N}\rho \left(e\left(i\right)\right),$$

where ρ(·) is the cost function of e(i). After *K* hypotheses are calculated, we aggregate the different estimated points ${\hat{x}}_{t,i}$ through weight $\omega_{k}$. In order to aggregate the point ${\hat{x}}_{t,i}$, we adopt a weighted mean strategy through:

(12) $${\hat{x}}_{t,agg}=\frac{\sum_{k=1}^{K}\omega_{k}{\hat{x}}_{t,i}^{k}}{\sum_{k=1}^{K}\omega_{k}}.$$

Our final robust motion estimation algorithm is shown in Algorithm 2. The correspondence points are sorted by Equation (5). We select *m* data points randomly from the front of the sorted feature points $u_{n}$. Then, a hypothesis $H_{k}$ is generated by minimizing Equation (3) and is verified by Algorithm 1. We record the number of inliers, which is calculated by Equation (9). If the termination criterion of SRPT reaches, we stop generating a hypothesis and the three best hypotheses $H_{1},H_{2},H_{3}$ are recorded. If not, the hypothesis will be generated by selecting *m* data points in the set of $u_{n+1}$. Finally, the inliers in the best hypothesis will be aggregated by Equation (12) and the motion is refined by all of the aggregated inliers.

**Algorithm 2:** Robust motion estimation PASAC
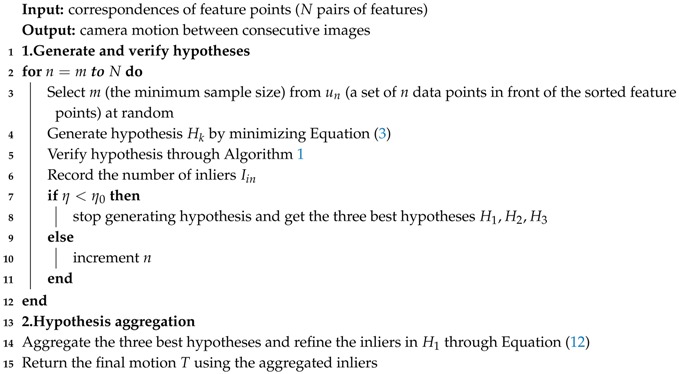


## 4. Results

In this section, we show the results of our approach for robust stereo visual odometry. We implemented our algorithm inside the Lib VISO2 (Library for Visual Odometry 2) [23] vision library and evaluated in the KITTI dataset [30] and the New Tsukuba dataset [31]. Our algorithm ran in real time and we carried out all experiments with an Intel Core-i5 (four cores @ 2.30 GHz) and 8 GB RAM. First, we describe two datasets in Section 4.1. Then, we show experiment results from datasets in both Section 4.2 and Section 4.3, respectively. Finally, the running time is described in Section 4.4.

### 4.1. Datasets

**Figure 7:**
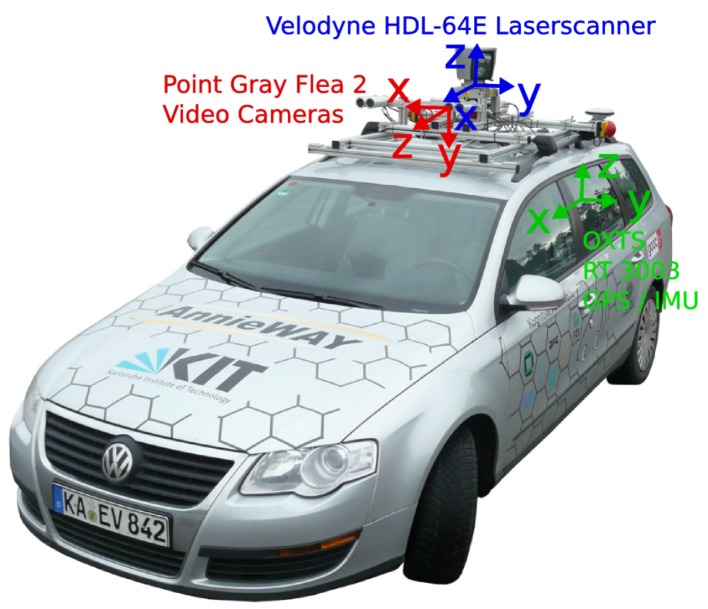
KITTI car. Equipped with two stereo cameras, GPS and a Velodyne laser scanner.

The KITTI odometry dataset provides 20 sequences recorded from cars driven in urban and rural areas and on highways. As illustrated in Figure 7, these cars are equipped with two stereo cameras, GPS and a Velodyne laser scanner. The first 11 sequences with ground truth ego-motion are used for training, without which the rest ones are used to evaluate. These cars speed up to 80 km/h and travel about 20,000 m. The dataset contains 23,201 image frames, taken at 10 fps with a resolution of 1241×376 pixels. The images are rectified and the calibration parameters of the cameras are provided. In order to evaluate different methods, translation and rotation errors are normalized with respect to path length and speed.

**Figure 8:**
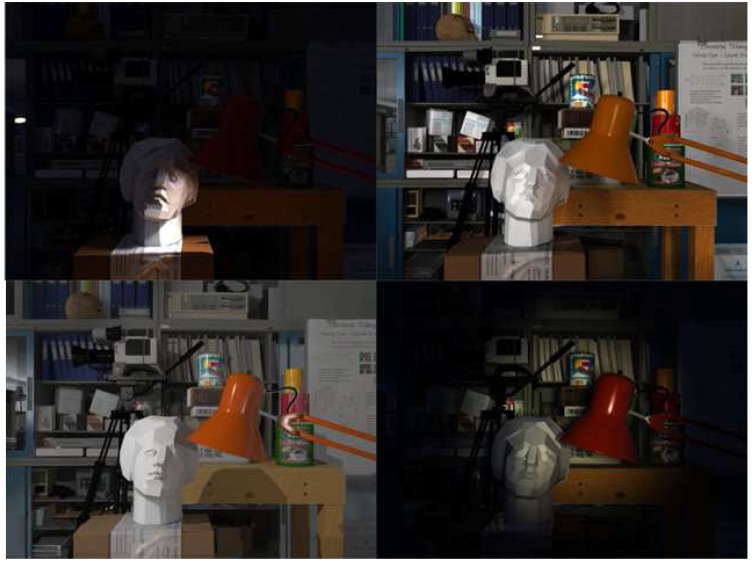
Four illumination conditions: upper-left: lamps; upper-right: fluorescent; lower-left: daylight; lower-right: flashlight.

The New Tsukuba dataset is a dataset for stereo matching and camera tracking evaluation. The dataset is a virtual sequence generated by the software Autodesk Maya 2012 (Autodesk, San Rafael, CA, USA). This dataset contains four different versions of the illumination conditions: daylight, fluorescent lighting, flashlight and desk lamps (seen in Figure 8). The virtual cameras navigate into an indoor laboratory and capture images with a resolution of 640×480 pixels at 30 fps.

### 4.2. Evaluation on the KITTI Dataset

**Figure 9:**
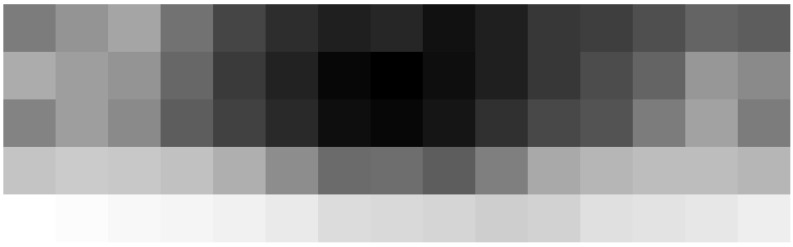
Distribution of reprojection error. Lower values correspond to darker colors. The error increases in all directions from the minimum, which is close to the image center.

It can be seen in Figure 9 that the inaccurate camera calibration affects the distribution of the reprojection error, as described in [32]. For the sake of adding some robustness against calibration errors, we use a feature weighting scheme when calculating the ego-motion parameters, which can be expressed as:

(13) $$\omega_{i}=\left(\right|u_{L}-u_{0}{\left|/u_{0}+0.05\right)}^{-1},$$

where $u_{0}$ is the camera horizontal principal point. Feature points closer to the principal point have higher weights in the optimization, whereas those closer to the image boundaries have lower weights.

**Figure 10:**
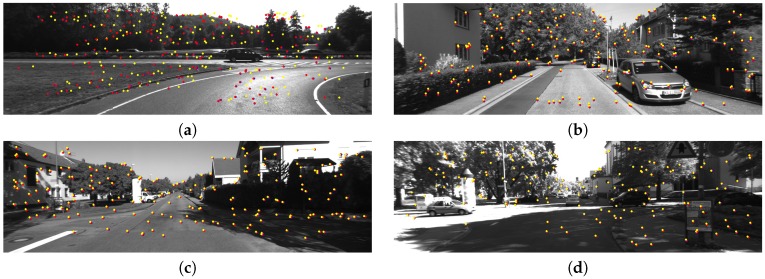
Feature points reprojection before and after hypothesis aggregation in four KITTI dataset sequences. Red points are reprojection from the ground truth motion, yellow and blue points are before and after hypotheses’ aggregation, respectively. (**a**) KITTI 01; (**b**) KITTI 03; (**c**) KITTI 05; (**d**) KITTI 08.

In order to get a better understanding of the procedure of hypothesis aggregation, Figure 10 shows the results of the distribution of feature points before and after we use hypothesis aggregation. Standard RANSAC always chooses the hypothesis with the largest support and discards other estimation. In Figure 10, the yellow feature points are the inliers selected by standard RANSAC, while the blue feature points are refined by our method. Using the ground truth ego-motion and disparity, we compute the ground truth position denoted by the red points so as to compare the accuracy of the position of the feature points. As we can see, the refined position of feature points is closer to the ground truth. When wielding the refined position of feature points, we get a more accurate motion estimation.

**Figure 11:**
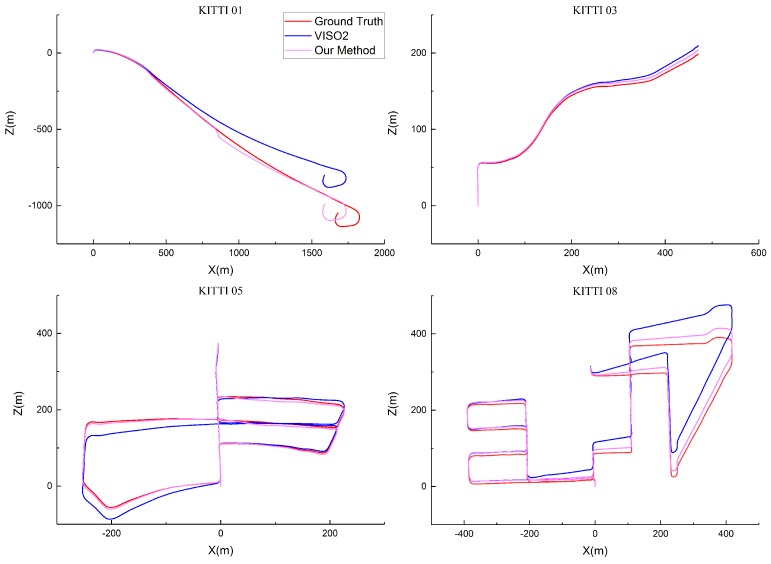
Trajectories on the sequences 01, 03, 05 and 08 of the KITTI dataset.

In Figure 11, we present a qualitative comparison of our trajectories, VISO2 and the ground truth. It is obvious that our method gets better results than VISO2 in sequences 01, 03, 05, 08.

**Figure 12:**
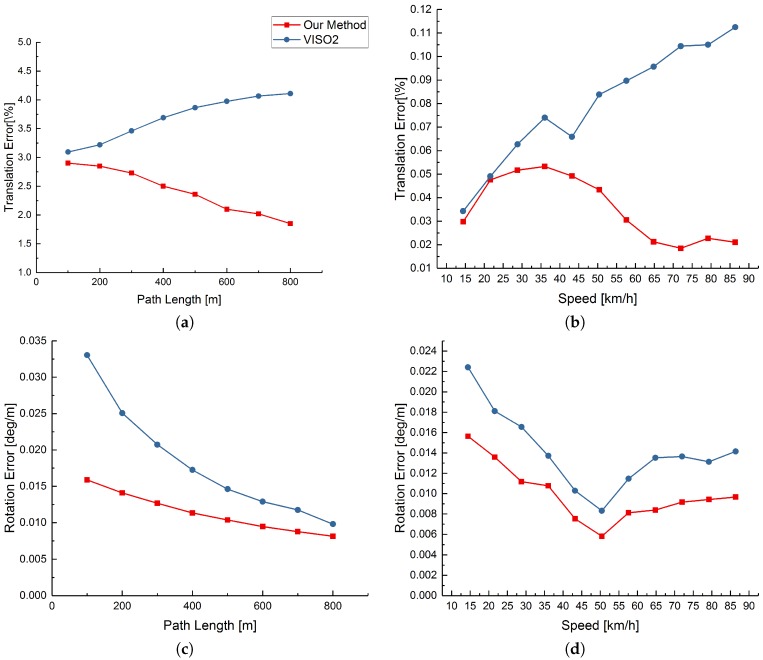
Average error on the KITTI dataset. Plots (**a**,**b**) refer to the average translation error for increasing path length and speed, respectively, while plots (**c**,**d**) refer to average rotation error.

Figure 12 indicates the average translation and rotation error, for increasing path length and speed, of our method and VISO2, referring to the 00 to 10 sequences of the KITTI training dataset. It is easy to see that our method gets better performance than VISO2 and also the bundle adjustment or loop closure detection is not used.

### 4.3. Evaluation on the New Tsukuba Dataset

**Figure 13:**
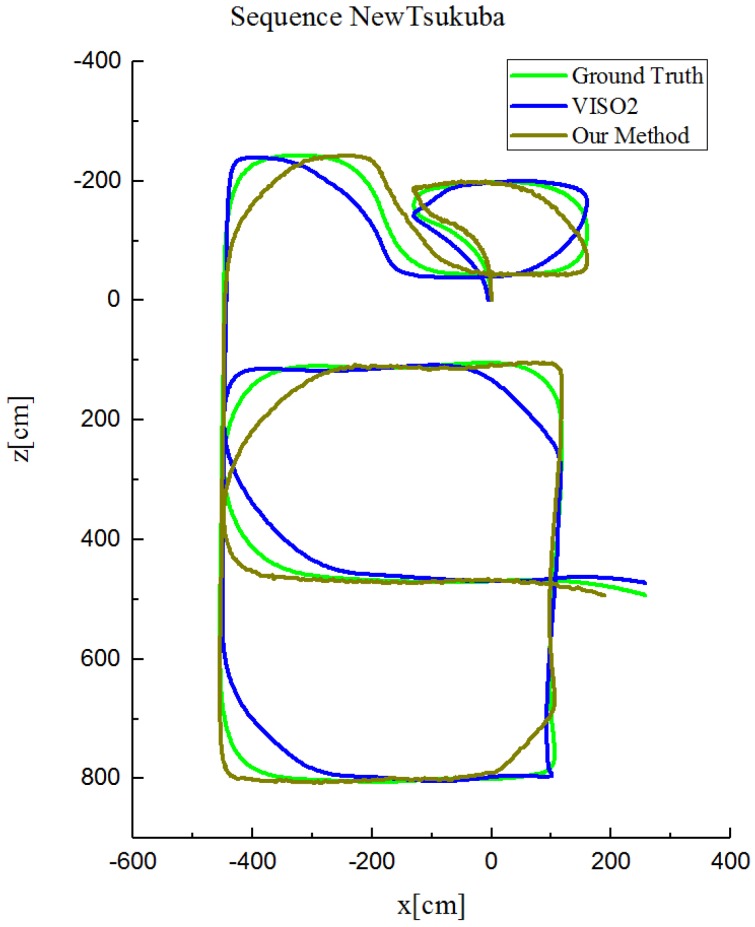
Trajectories on the daylight sequences of the New Tsukuba dataset.

In order to show the robustness of our method, we tested our method on the New Tsukuba dataset. Figure 13 shows the trajectories of both VISO2 and our method for the daylight sequence. Similar results are also obtained for the other three sequences. Our method achieves better results but not much. This is mainly because the camera moves very slowly in the Tsukuba dataset and fewer outliers appear even in standard RANSAC.

### 4.4. Running Time

For the purpose of comparing the efficiency of our method with standard RANSAC and PROSAC [27] (our method based on PROSAC), in each frame, we record the ratio of inliers ($I_{in}$), the total generated hypotheses ($K_{H}$), the verified points in each hypothesis ($V_{H}$), and the total time cost of the process of motion estimation (T). By using LibVISO2 with standard RANSAC, the total hypotheses needed to be generated are fixed to 200. The results are shown in Table 1. We tested six datasets in KITTI datasets and, in each sequence, the average $I_{in}$, $K_{H}*V_{H}$, and T were calculated. Our methods have a larger inlier ratio than RANSAC and PROSAC [27], which is confirmed in the fourth column of Table 1. The main reason is that more inliers can be found by our priori-based hypothesis generation strategy even when the circle matching is not good enough. Standard RANSAC needs to explore more hypotheses to find more inliers. Our methods, on average, have the smallest number of $K_{H}*V_{H}$, which means that our methods only need to generate less hypotheses and verify less points to achieve more accurate motion estimation. As seen in the last column, our method is seven times faster than RANSAC and two times faster than PROSAC, even after the procedure of hypothesis aggregation.

## 5. Conclusions

In this paper, we present a robust stereo visual odometry using an improved RANSAC-based method PASAC that makes the procedure of motion estimation much faster and more accurate than standard RANSAC. The main purpose is to strengthen RANSAC’s capability to deal with low calculating speed and accuracy when the inlier ratio is too low. It turns out that a robust stereo visual odometry can calculate ego-motion of the mobile robot very fast and accurately. The proposed method is evaluated in the KITTI and the New Tsukuba dataset. The experimental results demonstrate that the proposed method is more accurate and faster than RANSAC.

Our method follows a traditional feature-based visual odometry pipeline. Feature points are detected and matched by the circle matching. The ego-motion of the mobile robot can be calculated by the LM algorithm on the basis of minimizing the reprojection error. The main contribution is the robust motion estimation methods for visual odometry. In the process of robust motion estimation, we made an improvement in RANSAC by three steps, which are hypotheses generation, hypotheses evaluation and hypothesis aggregation: (1) the feature points are selected to generate hypotheses on the order of ages and similarities; (2) hypotheses are evaluated by SRPT without verifying all of the input data points; and (3) the three best hypotheses are aggregated to generate a better hypothesis. Thus, the proposed method calculates very quickly and accurately. For this reason, our robust visual odometry is very suitable to be used for mobile robot localization in outdoor and indoor environments.

In our future work, we would like to use some other robust features (e.g., line features) and add additional sensors (IMU) to improve the accuracy of the localization. In addition, we would like to extend our VO system to the VSLAM system with loop closure detection.

## Figures and Tables

**Table:** 

	
(**a**)	(**b**)
	
(**c**)	(**d**)

**Table:** 

	
(**a**)	(**b**)
	
(**c**)	(**d**)

**Table 1:** Comparison of efficiency of the estimation algorithm. Average computational time for a single frame on an Intel-i5 2.30GHz CPU, four cores are used.

KITTI Dataset	I${}_{in}\;\left(\%\right)$			K${}_{H}*$V${}_{H}$			T (ms)		
	RANSAC	PROSAC	Our Method	RANSAC	PROSAC	Our Method	RANSAC	PROSAC	Our Method
00	75.6	85.2	**89.8**	41520	1520	**845**	72.5	15.3	**8.5**
01	76.4	88.6	**92.2**	34508	1400	**950**	70.3	17.5	**10.5**
02	80.4	86.5	**95.5**	25004	**750**	788	65.2	**12.3**	15.2
03	78.8	84.4	**90.3**	32075	1004	**850**	67.5	21.2	**9.5**
04	82.7	82.1	**89.2**	30258	950	**787**	62.3	20.1	**6.5**
05	85.5	89.3	**93.1**	33382	1045	**920**	51.5	16.5	**5.7**
Avg.	79.9	86.0	**91.7**	32791	1112	**857**	64.9	17.2	**9.3**

## Abbreviations

The following abbreviations are used in this manuscript:

VO	Visual Odometry
VSLAM	Visual Simultaneous Localization and Mapping
RANSAC	Random Sample Consensus
SPRT	Sequential Probability Ratio Test
KLT	Kanade–Lucas–Tomasi Feature Tracker
SAD	Sum of Absolute Differences
LM	Levenberg–Marquardt
